# The detection of Japanese encephalitis virus in Megachiropteran bats in West Kalimantan, Indonesia: A potential enzootic transmission pattern in the absence of pig holdings

**DOI:** 10.1016/j.ijppaw.2021.03.009

**Published:** 2021-03-22

**Authors:** Ajib Diptyanusa, Elisabeth Siti Herini, Soedarmanto Indarjulianto, Tri Baskoro Tunggul Satoto

**Affiliations:** a Doctoral Study Program of Health and Medical Sciences, Faculty of Medicine, Public Health and Nursing, Universitas Gadjah Mada, Indonesia; b Department of Parasitology, Faculty of Medicine, Public Health and Nursing, Universitas Gadjah Mada, Indonesia; c Department of Child Health, Faculty of Medicine, Public Health and Nursing, Universitas Gadjah Mada, Indonesia; d Department of Internal Medicine, Faculty of Veterinary Science, Universitas Gadjah Mada, Indonesia

**Keywords:** Japanese encephalitis, Zoonosis, Transmission, Bats, Chiroptera, Indonesia

## Abstract

The West Kalimantan province in Borneo island, Indonesia belongs to endemic area of Japanese encephalitis (JE) that accounts for approximately 30% of total cases yearly. As the presence of pig holdings is uncommon in West Kalimantan, another reservoir host might have played a role in the local transmission of JE virus in this area. Current study aimed to identify the potential role of bats in the local transmission of JE by performing molecular detection of JE virus in bats and mosquitoes using RT-PCR. Sample collection was performed in 3 districts in West Kalimantan, covering 3 different ecosystems: forest, coastal, and residential areas. Bat collection was performed using mist net and harp net, while mosquito collection was carried out using animal-baited trap and human landing collection. A total of 373 blood samples from bats were tested for JE virus, among which 21 samples (5.6%) showed positive results, mainly from *Cynopterus brachyotis* (lesser short-nosed fruit bat) found in residential areas. Out of 53 mosquito pools, 3 JE-positive pools of *Culex tritaeniorhynchus* and *Cx. vishnui* were collected at the same location as JE-positive bats. Current study showed the first evidence of JE virus detection in several species of Megachiropteran bats in Indonesia, demonstrated the potential role of frugivorous bats in local transmission of JE in West Kalimantan. More aggressive measures are required in JE risk mitigation, particularly in initiating JE vaccination campaign and in avoiding disruption of bats’ natural habitats through changes in land-use.

## Highlights

• First evidence of JE virus detection in Megachiropteran bats in Indonesia.
• Molecular detection of JE virus using RT-PCR instead of using antibodies.
• Collection of JE-positive bats and mosquitoes at the same site.
• Involvement of bats in JE transmission cycle in the absence of pig holdings.

## Graphical abstract

**Figure:**
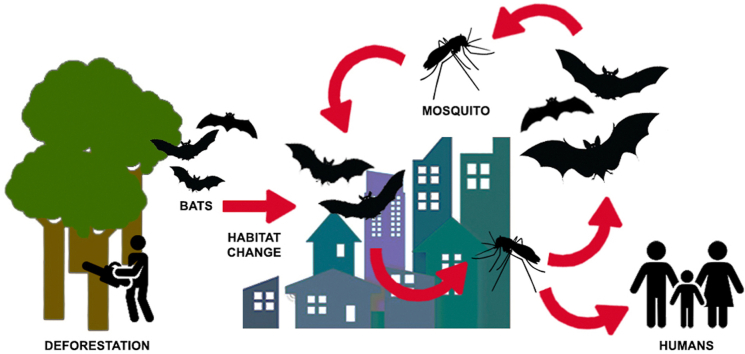

## 1 Introduction

Japanese encephalitis (JE) virus is one of the most common cause of viral encephalitis in Asia (Solomon et al., 2003). Classic transmission cycle of JE virus involves pigs as reservoir or amplifying hosts, while the virus itself is transmitted to humans through mosquito bites, mainly *Culex* species (Choe et al., 2018; Yun and Lee, 2014). Although Indonesia belongs to endemic area of JE, routine screening in individuals presenting with acute encephalitis syndrome has been lacking due to limited data on true prevalence of the disease in many provinces (Ma'roef et al., 2020). Sporadic cases have been reported in a number of areas in the past 20 years, including Bali and several cities in Java and Kalimantan (Borneo) island (Kari et al., 2006; Konishi et al., 2009; Liu et al., 2010; Maha et al., 2009). In the year 2016, the JE sentinel surveillance on children presenting with acute encephalitis syndrome conducted in 11 provinces in Indonesia revealed JE incidence of 15.2% (MoH of Republic of Indonesia, 2017). Reported JE cases from West Kalimantan province alone accounted for approximately 20%–30% of total JE cases yearly (MoH of Republic of Indonesia, 2019). Interestingly, pig farming practices in West Kalimantan are uncommon, hence classic enzootic transmission cycle of JE that involves pigs as amplifying hosts in this area is less likely to occur.

In the past few years, the importance of bats as potential reservoir hosts for arboviruses including JE virus has been more frequently mentioned (Calisher et al., 2006; Fagre and Kading, 2019). Antibodies against these arboviruses have also been detected in bats (Olson et al., 1983; van den Hurk et al., 2009), showing that bats might have had the capability as reservoir hosts for these viruses. The characteristic phenomenon of cross-species transmission of viruses (“spillover”) from bats to humans have also been increasingly recognized as a major pathway of transmission that affected human susceptibility to infection by these viruses (Letko et al., 2020; Wang and Anderson, 2019). Considering the peridomestic nature of several bat species that amplifies the possibility of zoonotic viral spillover (Luis et al., 2013), as well as the ability of *Culex* mosquitoes in switching blood feeding host preference (Mwandawiro et al., 2000), the authors proposed uncommon JE transmission cycle involving bats as reservoir hosts that occurred in West Kalimantan province in Indonesia. Although the potential role of bats in the transmission cycle of JE virus has been described elsewhere (Yun and Lee, 2014), studies have been limited to identification of circulating antibodies against JE virus in bats particularly in the older group of Microchiroptera (microbats) (Banerjee et al., 1988; Cui et al., 2008; Jiang et al., 2015; Miura et al., 1970). Current study aimed to detect JE virus in both bats and mosquitoes collected in West Kalimantan province in Indonesia to support the idea of potential role of bats in the transmission of JE virus in study area. The distribution of bat species along with species diversity were also investigated to describe the probability of viral spillover to humans.

## 2 Material and methods

Current study was a part of the integrated national-scale disease vector and reservoir surveillance program of Indonesia (*Rikhus Vektora*) conducted by the Ministry of Health (MoH) of Republic of Indonesia from year 2015 through 2018. Study locations were determined based on the previous reports of human clinical cases of JE by the local health office (MoH of Republic of Indonesia, 2019). Sampling of bats and mosquitoes was purposively performed in 3 districts in West Kalimantan (Kayong Utara, Sambas, Ketapang), covering 3 different ecosystems: forest, coastal, and residential areas. Forest was defined as plantation area of either primary or secondary origin. Coastal area was described as either beach, marshes, or tidal areas. Residential area was defined as residential environment that consists of more than one housing unit. Sample collection was performed for 12 h (from 6 p.m. to 6 a.m.) for 3 consecutive days. Location coordinates and environmental factors including temperature, relative humidity and wind velocity were recorded hourly during specimen collection.

### 2.1 Ethics statement

The performance of capture, blood specimen collection, maintenance, and release of bat samples were conducted according to the Animal Welfare Act, 2006 regarding management of wild animals (AWA, 2006). The approval for ethical principles regarding current study was granted by the Medical and Health Research Ethics Committee (MHREC) of the Faculty of Medicine, Public Health and Nursing, Universitas Gadjah Mada (Ref. No. KE/FK/0339/EC/2020).

### 2.2 Bat collection

Bat collection was performed using mist net and harp net (Struebig and Sujarno, 2006; Thomas and West, 1989). Pregnant or lactating bats with dependent young bats were released and were excluded from the study. Anesthesia was given to the captured bats prior to species identification and blood collection. Bat species and sexual characteristics were recorded using the appropriate identification key for Asian bats (Corbet and Hill, 1992; Srinivasulu et al., 2010). Blood samples were collected from brachial vein according to the procedure mentioned in another study (Eshar and Weinberg, 2010), and were put onto 125 μl FTA card (Whatman, Merck, Germany). The FTA cards were kept in room temperature (20 °C–25 °C) prior to RNA extraction. Blood samples from all collected bat species underwent molecular testing for JE virus.

### 2.3 Mosquito collection

Mosquito collection was carried out using animal-baited trap and human landing collection (Service, 1993; Taboada, 1967). Morphological identification key for mosquito species (Rattanarithikul and Panthusiri, 1994) was used to determine species of collected mosquito samples. Collected mosquitoes were then put into microtube filled with 500 μl of RNA later reagent (RNAlater, Thermo Fisher, USA) to preserve viral RNA. A single microtube was filled with “pooled” mosquitoes containing 1 to 25 mosquitoes. Each microtube was filled with mosquitoes grouped according to mosquito species, collection method, and time of collection. Mosquito pools were kept in 4 °C prior to further examination.

### 2.4 RNA extraction

FTA cards containing collected bat blood samples were cut out into 3 pieces of paper with 2 mm diameter each. These cut paper pieces were put into microplate and immersed with 100 μl of RNA rapid extraction solution (MagMAX, Thermo Fisher, USA) until blood samples were completely dissolved. Reagent preparation and RNA extraction procedures were performed according to manufacturer's instructions for MagMAX Viral RNA Isolation Kit (Thermo Fisher, USA).

Pooled mosquito samples were retrieved from microtubes containing RNAlater solution and were dissected, leaving only the head and thorax parts. These head-and-thorax preparations were put into new microtubes filled with 500 μl of PBS solution. Mosquitoes were grinded using pellet pestle. Total RNA extraction from pooled mosquitoes was carried out according to RNAeasy Mini Kit (Qiagen, Germany) manufacturer's instructions. Extracted RNA from both bat samples and mosquito pools were kept in −80 °C prior to molecular testing for JE virus.

### 2.5 Molecular detection of JE virus

The detection of JE virus in blood samples and mosquito pools was performed using reverse transcriptase polymerase chain reaction (RT-PCR) using JE-specific primers of 5′-AGA GCG GGG AAA AAG GTC AT-3’ (forward) and 5′-TTT CAC GCT CTT TCT ACA GT-3’ (reverse) targeting NS3 gene of JE virus (Igarashi et al., 1994; Tanaka, 1993). The PCR reaction was performed as described previously in another study (Gao et al., 2013). Electrophoresis was conducted in PCR products using 2% agarose gel. Band visualization of PCR products by SYBR safe DNA gel staining (Invitrogen, USA) at 162 bp was considered positive.

### 2.6 Data analysis

Coordinates of sample collection sites were plotted into the map using the software ArcGIS ver. 9.2 (Esri, New York). Average nearest neighbor (ANN) analysis was performed to identify the distribution pattern of JE vectors and reservoirs according to the nearest neighbor ratio (R) (Jacquez et al., 2006) values as follows: R of <1 (clustered distribution), R of 1 (random distribution), R of >1 (dispersed distribution) (Aziz et al., 2012). Buffer analysis was also carried out to visualize overlaps between mosquito and bat flight ranges. Estimated maximum *Culex* flight range was defined as 2 km (Ciota et al., 2012), while estimated average hunting ranges for Megachiropteran and Microchiropteran bats were defined as 30 km and 10 km, respectively (Hodgkison et al., 2004; Hutson et al., 2001). Both qualitative and quantitative data were analyzed using SPSS ver. 18.0 (SPSS, IL, USA). Appropriate bivariate analysis was performed to identify association between variables. Variables were considered as statistically significant if they demonstrated p-value of <0.05 (two-sided). Species diversity of bats was evaluated using the Shannon index (H) according to the following values (Magurran, 2004): H of <1.5 (low diversity), H of 1.5–3.5 (high diversity), H of >3.5 (very high diversity).

## 3 Results

A total of 554 bats were captured in study sites. After initial screening on pregnant or lactating bats with dependent young bats, 181 captured bats were released and were excluded from the study. Finally, 373 blood samples collected from bats underwent molecular detection of JE virus in the study. Among 373 bat samples, 21 samples (5.6%) were found positive for JE virus. Positive samples were collected from the following species: *Balionycteris maculata* (spotted-winged fruit bat; 3 samples), *Cynopterus brachyotis* (lesser short-nosed fruit bat; 11 samples), *C. sphinx* (greater short-nosed fruit bat; 1 sample), *Eonycteris spelaea* (lesser dawn bat; 3 samples), and *Macroglossus minimus* (lesser long-tongued fruit bat; 3 samples). Characteristics of collected bat samples are shown in Table 1. Both juvenile and adult bats were found in similar proportion in JE-positive group. Male to female ratio was 1:1 in the same group. Bats infected with JE virus had the tendency to have smaller weight than that of uninfected ones, although the difference was not found to be statistically significant. All of the JE-positive bats belonged to suborder Megachiroptera (megabats), among which 10 (47.6%) samples were captured in the forest area.

**Table 1:** Basic characteristics of collected bat samples based on JE virus test results.

Parameter	Total^a^ N = 373	JE-negative^a^ N = 352	JE-positive^a^ N = 21	*P-value*
Estimated age				
Juvenile	131 (35.1)	120 (34.1)	11 (52.4)	0.088^c^
Adult	242 (64.9)	232 (65.9)	10 (47.6)	
Sex				
Male	208 (55.8)	197 (56.0)	11 (52.4)	0.748^c^
Female	165 (44.2)	155 (44.0)	10 (47.6)	
Weight (gram)^b^	32 (3–74)	31 (3–74)	31 (10–40)	0.280
Collection sites				
Forest	138 (37.0)	128 (36.4)	10 (47.6)	0.354^d^
Coastal area	138 (37.0)	133 (37.0)	5 (23.8)	0.248^d^
Residential area	97 (26.0)	91 (25.9)	6 (28.6)	0.799^d^
Suborder				
Megachiroptera	348 (93.3)	327 (92.9)	21 (100)	N/A
Microchiroptera	25 (6.7)	25 (7.1)	0	

a presented in frequency (%).

b presented in median (min-max); Mann-Whitney test.

c Chi square test.

d Fisher exact test.

In general, as many as 22 bat species were included in the study. Majority (72.9%) of total bat samples belonged to genus *Cynopterus*, particularly *C. brachyotis*. The number of tested bat samples collected in the forests and in coastal areas was similar, yet the diversity index differed (Table 2). Low species diversity was observed in the forest, showing domination of a single species (*C. brachyotis*) in the similar ecosystem. On the contrary, highest diversity index was seen in residential areas (H value of 1.74).

**Table 2:** Collected bat species according to collection sites and species diversity.

No.	Bat species	Collection sites			Total	JE-positive
		Forest	Coastal area	Residential area		
1.	*Balionycteris maculata*	7^a^	–	–	7	3
2.	*Cynopterus brachyotis*	101^a^	71^a^	49^a^	221	11
3.	*C. horsfieldii*	3	2	3	8	–
4.	*C. minutus*	5	8	6	19	–
5.	*C. sphinx*	12^a^	8	3	23	1
6.	*C. titthaecheilus*	–	–	1	1	–
7.	*Eonycteris major*	–	–	1	1	–
8.	*E. spelaea*	–	23^a^	9^a^	32	3
9.	*Glischropus tylopus*	–	–	1	1	–
10.	*Hesperoptenus* sp.	–	1	–	1	–
11.	*Hipposideros cineraceus*	1	–	–	1	–
12.	*H. galeritus*	1	–	–	1	–
13.	*Kerivoula* sp.	1	–	–	1	–
14.	*Macroglossus minimus*	3^a^	20^a^	10^a^	33	3
15.	*Megaerops wetmorei*	1	–	–	1	–
16.	*Myotis muricola*	–	–	7	7	–
17.	*Penthetor lucasi*	1	1	–	2	–
18.	*Rhinolophus borneensis*	–	–	1	1	–
19.	*Rh. trifoliatus*	1	–	–	1	–
20.	*Saccolaimus saccolaimus*	–	4	–	4	–
21.	*Taphozous* sp.	–	–	6	6	–
22.	*Tylonycteris* sp.	1	–	–	1	–
	Total	138	138	97	373	21
	Shannon diversity index (H)	1.13	1.49	1.74		

a some samples were found positive for JE virus.

Fifty-three mosquito pools collected from a total of 12 mosquito species were included in current study. These JE-tested mosquitoes belong to the genera that have previously been reported to be natural vector for JE virus in Indonesia (*Aedes, Coquillettidia, Culex, Mansonia*). All of collected *Anopheles* mosquitoes underwent *Plasmodium* sporozoite detection by the MoH of Republic of Indonesia and were not studied for JE virus and hence not discussed in current study. As shown in Table 3, *Culex tritaeniorhynchus* was the most dominant mosquito species collected from the study sites (234/984; 23.8%), followed by *Mansonia uniformis* (209/984; 21.2%) and *Cx. vishnui* (198/984; 20.1%). Human landing collection was the most successful method used in mosquito collection (44/53; 83.0%). Among 53 tested mosquito pools, 3 pools (5.7%) were found positive for JE virus: *Cx. tritaeniorhynchus* (1 pool), *Cx. vishnui* (2 pools). None of the JE-positive mosquito pools was collected in the forests. Assumptions for bivariate analysis were not fulfilled for mosquito samples, hence statistical analysis was not performed in the mosquito group.

**Table 3:** Number of pooled mosquito species underwent molecular testing according to collection sites.

No.	Mosquito species	Collection sites			Total	JE-positive
		Forest	Coastal area	Residential area		
1.	*Aedes andamanensis*	–	2	1	3	–
2.	*Coquillettidia crassipes*	1	–	–	1	–
3.	*Culex gelidus*	1	2	2	5	–
4.	*Cx. quinquefasciatus*	1	–	1	2	–
5.	*Cx. sinensis*	2	–	–	2	–
6.	*Cx. sitiens*	2	–	–	2	–
7.	*Cx. tritaeniorhynchus*	7	3^a^	5	15	1
8.	*Cx. vishnui*	4	4^a^	1^a^	9	2
9.	*Mansonia bonneae*	1	1	–	2	–
10.	*Mn. dives*	1	–	–	1	–
11.	*Mn. indiana*	–	–	1	1	–
12.	*Mn. uniformis*	4	3	3	10	–
	Total	24	15	14	53	

a one pool each was found positive for JE virus.

Table 4 described the results of environmental factors recorded at the time of sample collection in study sites. The lowest recorded temperature was 24.1 °C that was observed in the forests, while the highest temperature (32.0 °C) was observed in residential areas. Lowest humidity (67.9%) was observed in coastal areas. Coastal areas demonstrated the highest documented value of average wind velocity (15.8 m/s). Mean differences were observed in temperature, humidity and wind velocity among all three ecosystems (*P* < 0.05).

**Table 4:** Recorded environmental factors at sample collection sites.

Parameters^a^	Collection sites		
	Forest	Coastal area	Residential area
Temperature (^0^C)			
Minimum^b^	25.9 ± 0.7	27.1 ± 1.7	27.0 ± 1.0
Maximum^b^	29.4 ± 1.6	30.2 ± 1.1	30.5 ± 1.3
Humidity (%)			
Minimum^b^	76.5 ± 6.3	76.3 ± 4.1	72.1 ± 3.9
Maximum^b^	82.7 ± 5.6	84.7 ± 4.9	83.5 ± 4.0
Wind velocity (m/s)			
Maximum^b^	1.5 ± 1.0	5.1 ± 3.5	4.5 ± 1.6

a presented in mean ± SD.

b observed mean differences; p < 0.05 (ANOVA).

**Fig. 1:**
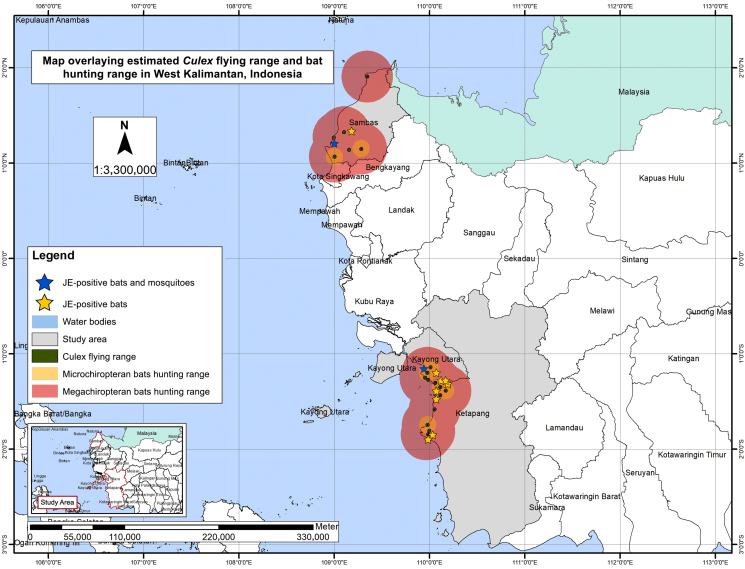
Buffer map overlaying estimated flight range of *Culex* mosquitoes and hunting ranges of bats.

Overall, buffer map was developed using coordinates of sample collection, PCR results for JE virus, and the estimated flight ranges of mosquitoes and bats (Fig. 1). A total of 15 bat collection sites yielded captured bats with positive results for JE virus. All of the JE-positive mosquito pools was also collected at the same collection sites as those of infected bats. Overlapping flight ranges of JE-infected *Culex* mosquitoes and the bats was observed. The individual ANN analysis of JE-positive bats in Kayong Utara, Sambas, and Ketapang districts resulted in similar R value of <1 (*P* < 0.001) that demonstrated clustered distribution.

## 4 Discussion

Current study showed the first evidence of JE virus detection in several species of Megachiropteran bats in Indonesia. Potential role of bats in the transmission cycle of several arboviruses has been put into the light in the past decade (Calisher et al., 2006; Fagre and Kading, 2019), particularly through the demonstration of circulating antibodies against these arboviruses in serum samples from bats (Cui et al., 2008; Irving et al., 2020; Olson et al., 1983). Antibodies against JE virus have been detected in bats belonged to suborder Microchiroptera, including *Hipposideros armiger*, *H. pomona*, *H. speoris*, *H. bicolor*, *H. cineraceus*, *Rhinolophus comutus*, *R. macrotis*, *R. rouxi*, *R. ferrumequinum*, *Vespertilio superans*, *Myotis macrodactylus,* and *Miniopterus schreibersii* (Banerjee et al., 1988; Calisher et al., 2006; Cui et al., 2008; Jiang et al., 2015; Miura et al., 1970). However, data regarding molecular detection of JE virus in bats have been very limited until recent identification of JE virus in *Pteropus* sp. in Indonesia (Saepulloh et al., 2016). While antibodies were mostly detectable and vastly studied, the presence of circulating JE virus in bats has been assumed to be under detectable level as infection is always asymptomatic (Cui et al., 2008). Still, the detection of JE virus in blood samples from bats through PCR remains vital, as the virus is thought to be transmitted to bats from mosquitoes bites (Fagre and Kading, 2019), and it is possible that mosquitoes will become infected after taking bloodmeal from infected bats.

As shown in current study results, several mosquito pools belonged to *Cx. tritaeniorhynchus* and *Cx. vishnui* were found to be positive for JE virus. These mosquito species belong to major vectors of JE virus particularly in Asia (Liu et al., 2018; Toma et al., 2000). Female *Culex* mosquitoes are often zooanthropophilic, which means natural host preferences of animal hosts (particularly pigs) may shift to humans or other animal hosts in the absence of these preferred animal hosts (Mwandawiro et al., 2000). In West Kalimantan, the absence of pigs as natural preferred hosts for *Culex* female mosquitoes might have altered their behavior, hence we propose the possibility of mosquito blood feeding from bats. Such concept has been demonstrated in *Culex* mosquitoes collected nearby caves in Thailand (Tiawsirisup et al., 2012). Moreover, alterations in environmental factors affecting natural breeding sites or habitat can also contribute to changes in mosquito distributions, and later, their behavior (Ciota et al., 2014; Connor and Bunn, 2017). The main breeding habitat of *Cx. tritaeniorhynchus* as the major vector of JE virus is typically rice field with short and sparse vegetation (Erlanger et al., 2009; Keiser et al., 2005), although variations in breeding sites located into more urban areas have been reported possibly due to intense urbanization (Liu et al., 2018). Modified land-use creates habitat fragmentation that might have played a role in the expansion of anthropophilic mosquitoes as well as forest-dwelling bats into residential areas, thus increasing the risk of human disease transmission (Hassell et al., 2017; Mayi et al., 2020; Nunes et al., 2017). Current study results showed that 5 of 15 *Cx. tritaeniorhynchus* pools were collected in residential areas, although all of these mosquito pools yielded negative results for JE virus. However, such findings were quite alarming considering that some of the JE-infected *C. brachyotis*, *E. spelaea*, and *M. minimus* were captured from the same residential areas where *Cx. tritaeniorhynchus* were collected. These bat species’ natural habitats include tropical rainforests, agricultural areas, or caves (Mikail et al., 2017), and are not commonly found in residential areas. In contrast to that of mosquitoes, responses to land-use changes in bats are not commonly uniform and more likely to depend on the type of previous natural habitat where bats reside (Yoh et al., 2020), for example forest-dwelling bats are more susceptible to habitat disruption from urbanization.

Due to changes in global temperature, arboviral disease transmission is most likely to be affected (Patz et al., 2005). Under laboratory condition, JE virus can maintain its existence in bats for over 15 weeks or longer in colder temperature (temperature lower than 24 °C), a characteristic commonly called as “viral overwintering” (Calisher et al., 2006; Sulkin and Allen, 1974). Data regarding relationship between temperature and *Culex* competence in JE virus transmission is lacking, however, early study demonstrated that JE virus transmission rate was faster in temperature of 28 °C or higher, and that infection rate was much slower in temperature less than 26 °C (Takahashi, 1976). This might be due to the fact that higher temperature can cause faster mosquito development time and reduced extrinsic incubation period of JE virus in mosquito vectors, affecting the JE virus transmission (Tian et al., 2015; Wang et al., 2014). A mathematical model showed an increased rate of JE virus transmission by 14.4% for each 1 °C increase in temperature (Lin et al., 2017). Our study findings recorded various ranges of minimum and maximum temperature that mostly fell roughly from 25 °C to 32 °C, temperature range optimum for JE virus transmission in mosquitoes. Windy setting (wind velocity over 2 m/s) might have aided the flight range of infected *Culex* mosquitoes, increasing the risk of JE virus transmission especially in residential areas (Verdonschot and Besse-Lototskaya, 2014). These windblown, JE-infected mosquitoes will possibly be carried to bats’ natural habitats where they obtain blood feed and infect the bats. As some Megachiropteran bats tend to roost in large colonies (Brooke et al., 2000; Chruszcz and Barclay, 2002; Hahn et al., 2014), viral spillover is also likely to occur even with a single infected bat in the corresponding habitat (Letko et al., 2020; Plowright et al., 2015). Intraspecies viral spillover could greatly be increased in big roosts with small species diversity (Calisher et al., 2006; Wang and Anderson, 2019). This is supported by the finding of current study that showed the proportion of JE-infected bats was the highest in forest ecosystem with low diversity index. Furthermore, long-distance flight of Megachiropteran bats may also increase the chance of inter- and intraspecies spillover in other roosts, as illustrated in the overlapping flight ranges in current study. Laboratory demonstration of JE virus spillover among Megachiropteran bats, from bats to chicken and vice versa, was carried out in other study (Banerjee et al., 1984), showing that frugivorous bats can be potential reservoir hosts for JE virus maintenance (Mackenzie et al., 2016).

Aggressive One Health approach is essential in addressing issues in JE virus transmission through interdisciplinary research collaboration and policy-making for better disease control and prevention measures (Bidaisee and Macpherson, 2014; Wood et al., 2012). This study highlights the potential involvement of bats in the transmission cycle of JE virus in West Kalimantan where pig holdings are absent. Local government should be aware of zoonotic potential of JE virus, and that the transmission dynamics depend greatly on land-use and urbanization. Despite high prevalence of JE and the potentially serious neurological sequelae from the infection, routine JE vaccination program has yet to be implemented in West Kalimantan. Current study results should aid the decision making in the vital deployment of JE vaccination campaign, as well as in the expansion of JE surveillance the near future.

There are several limitations of current study that need to be addressed. Firstly, sample collection was only performed in a short period that might have been unable to adequately represent the actual JE infection rate or species abundance in the study sites. Secondly, sequencing analysis on JE-positive samples and mosquito blood meal identification were not performed, hence making it rather difficult to determine the same origin of JE virus in infected bats and mosquitoes. Nevertheless, current study highlighted the possibility of JE viral spillover and the increasing importance of identifying potential role of bats as reservoir hosts of JE virus where pig holding is absent.

## Disclaimer

Current study was a part of doctoral research conducted by the first author. This study also received a research funding from the Faculty of Medicine, Public Health and Nursing, 10.13039/501100012521Universitas Gadjah Mada (Ref. No. 303/UN1/FK-KMK/PP/PT/2020). Permission to publish the data was approved by the National Institute of Health Research and Development, Ministry of Health of Indonesia (Ref. No. 29011904-148). Some of the information in the tables were presented at the virtual Joint International Tropical Medicine Meeting (JITMM) in December 2020.

## Declaration of competing interest

None.
